# Differences in spatiotemporal pressure and performance between Chinese and German elite youth football players during matches

**DOI:** 10.3389/fpsyg.2025.1543287

**Published:** 2025-01-22

**Authors:** Yapu Liang, Tianhe Li, Hansi Xu, Peng Zhang

**Affiliations:** School of Strength and Conditioning Training, Beijing Sport University, Beijing, China

**Keywords:** spatiotemporal analysis, youth football, pressure, performance, tactics

## Abstract

**Introduction:**

In modern football, spatial and temporal pressure significantly influence player performance and tactical outcomes, particularly in youth competitions. This study aims to investigate the spatial pressure differences between Chinese and German U17 elite youth football teams, focusing on the ball-handler’s distance to the nearest defender (D).

**Methods:**

Video analysis was conducted to measure D across various match contexts, including scorelines (leading, tied, and trailing), game phases (passing and receiving), pass outcomes (successful and unsuccessful), and pitch zones. Statistical analyses were performed using non-parametric methods to compare the D under different conditions. The Mann–Whitney *U* test and Kruskal–Wallis *H* test were used to identify significant differences, with *post hoc* comparisons conducted where necessary.

**Results:**

Results show that the German team consistently maintained greater D than the Chinese team (*p* < 0.001, *d* = 0.463), highlighting their superior spatial management and tactical adaptability.

**Discussion:**

Greater D was associated with enhanced offensive flexibility and defensive stability, allowing the German team to create space effectively and maintain structural integrity under pressure. In contrast, the Chinese team’s smaller D suggested limitations in spatial utilization and higher defensive engagement risks. These findings underscore the importance of tactical training emphasizing spatial awareness and balanced pressure management, providing valuable insights for youth football development.

## 1 Introduction

In modern football, the time interval between transitions from attack to defense is becoming shorter, and the frequency of these transitions is increasing. High-intensity defensive tactics are considered a key component of future games (Nassis et al., 2020; Harper et al., 2021). Effective defending can significantly increase the pressure on the attacking side, leading to a higher “level of pressure” on players and preventing the opposition from organizing successful attacks, thereby reducing the likelihood of conceding goals for the defending team (Link et al., 2016). Defensive pressure primarily refers to the spatial pressure exerted by defenders on attacking players, aimed at limiting the actions available to attackers. It is often correlated with the performance of the defending team (Andrienko et al., 2017; Tenga et al., 2010).

Currently, high-pressing defensive tactics are gaining popularity, and research related to defending is continually evolving. Tengahe and Fernandez-Navarro, among others, have highlighted the differences in pressure exerted by various playing styles but have not provided conclusive results on the effectiveness of different defensive pressing behaviors (Fernandez-Navarro et al., 2020; Lepschy et al., 2021). At the same time, research on the attacking side is equally important, as it enables players to better manage their psychological state under pressure, thus maintaining their performance without being disturbed by mistakes (Chen et al., 2023). However, it remains difficult to determine the actual effect of pressure in football matches, with much of the existing literature offering subjective conclusions. Furthermore, research on pressure in youth football is scarce, with most studies focusing on adult athletes (Garcia et al., 2015; Coutinho et al., 2017). Understanding how defensive pressure manifests in youth football is crucial, as it not only influences tactical outcomes but also shapes the technical and psychological development of young players. Within a cross-cultural context, differing training methodologies may result in varied interpretations and applications of defensive strategies. Consequently, by examining defensive pressure in youth football, this study seeks to highlight its broader implications for player development and team performance. This study addresses these issues by focusing on how defensive pressure manifests in youth football across Germany and China, two countries with contrasting football cultures and training methodologies.

It is noteworthy that European football coaches place particular emphasis on training at the youth level (Forcher et al., 2024). Training scenarios are often set in small-sided games (SSGs) that simulate match-like conditions, aiming to enhance essential competitive skills in football (Herold et al., 2022). Therefore, training content in each session should cover approximately 70–80% of game-related scenarios. In contrast, the proportion of training dedicated to attacking and defensive situations in Chinese youth football is sometimes as low as 20%, a disparity that significantly impacts players’ understanding of the game (Gao et al., 2023).

Despite the growing focus on defensive pressure in football, there is limited understanding of how these metrics apply specifically to youth players, whose developmental and tactical approaches differ from adult athletes. Furthermore, cross-cultural analyses of defensive pressure remain underexplored, leaving gaps in how training philosophies and tactical systems shape defensive strategies in different footballing contexts. This study addresses these issues by focusing on how defensive pressure manifests in youth football across Germany and China, two countries with contrasting football cultures and training methodologies.

This study aims to analyze the characteristics of defensive pressure faced by youth players in different attacking scenarios during matches. Specifically, it seeks to compare the performance of Chinese and German youth football players, identify the characteristics of successful defending, and explore cross-cultural differences in defensive strategies. By addressing these objectives, the study aims to enhance the understanding of spatiotemporal dynamics in youth football and provide actionable insights for improving training practices.

To achieve this, the study focuses on two teams from contrasting football contexts: Germany, one of the world’s leading football nations with a high football participation rate, and China, a country that has made considerable efforts to develop football despite its relatively low football population density. This study hypothesizes that German youth players, with their emphasis on positional play and spatial management, will exhibit greater defensive distances compared to their Chinese counterparts. Conversely, Chinese players are expected to display higher intensity in defensive actions, reflecting their tactical focus on pressing and rapid transitions.

This study fills a critical gap in the literature by focusing on youth football, an area less explored compared to adult football. Adopting a cross-cultural perspective, it examines tactical differences and their roots in distinct training philosophies. The findings aim to quantify spatiotemporal defensive characteristics, highlight cross-cultural differences between Germany and China, and provide practical insights to enhance youth football training across diverse contexts.

## 2 Materials and methods

### 2.1 Materials

This study compared the Under-17 (U17) teams of the German Bundesliga club TSG 1899 Hoffenheim and the Chinese Super League club Beijing Guoan, both competing at the highest level of their respective national youth leagues. To maintain consistency and alignment with the research objectives, U17 matches were selected for analysis after initial video monitoring across multiple age groups (U15, U17, and U19). Two matches per team were chosen from four recorded games to ensure competitive balance and comparability. The German matches were sourced from the 2017 and 2018 German Youth Bundesliga (1st Division), while the Chinese matches were taken from the Chinese Football Association Super League (1st Division). All selected matches were home games to ensure consistency in the video recording setup. Matches were carefully selected to ensure competitive quality, with closely contested outcomes, high technical and tactical performance, and balanced competition. Efforts were made to minimize external factors, such as adverse weather conditions, that could affect match quality or recording clarity. To reduce seasonal variability, all matches were recorded within a two-month summer period.

While this study analyzed two matches per team to maintain balance, it is acknowledged that the limited sample size may restrict the generalizability of the findings. These matches were selected based on their competitive level and representativeness, ensuring that the data provided meaningful insights despite the constraints. Additionally, it is recognized that tactical variability, such as formations and individual roles, may influence the observed metrics. However, this study focuses on overarching defensive pressure characteristics rather than specific tactical nuances. These limitations highlight the exploratory nature of this research and lay the groundwork for future studies to expand the dataset and incorporate tactical factors for a more comprehensive understanding.

### 2.2 Protocol

**Figure 1:**
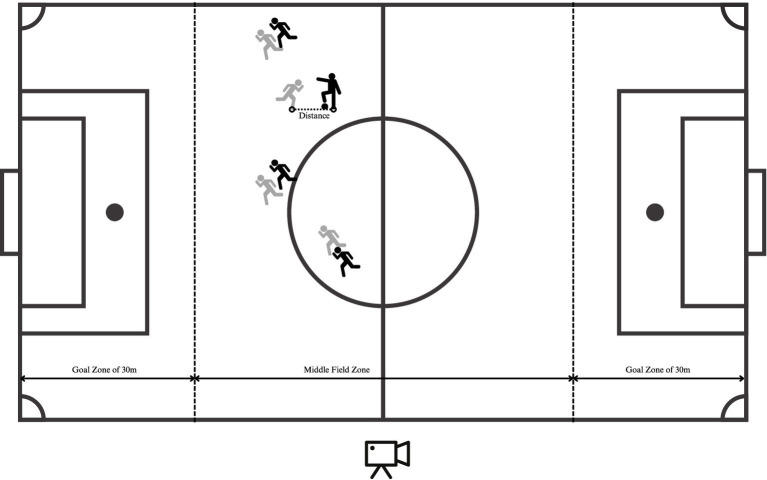
Schematic diagram of the experimental protocol.

Before the recording process, the pitch in each stadium was carefully calibrated to ensure precise spatial measurement. Pylons were strategically placed at specific points along the field’s boundary to aid in the calibration process. This setup ensured that the camera remained stationary throughout the game, eliminating any need for panning or zooming and thereby maintaining consistent image quality and perspective across all recordings. GoPro cameras (HERO14 Black) were employed for video recording, positioned 5 m away from both the centreline and the sideline at a fixed height, as shown in Figure 1. This configuration allowed for a comprehensive and unobstructed view of the pitch. The calibration process played a crucial role in linking the pixel-based coordinate system of the video to a predefined coordinate system on the field, enabling accurate spatial measurements of players’ movements and interactions.

### 2.3 Data processing

The data processing phase involved multiple steps to ensure the accuracy and reliability of the collected data. Video preprocessing was conducted to eliminate distortions. Utilized field calibration was performed on each frame to link pixel dimensions to real-world coordinates. A predefined field coordinate system was applied, enabling precise calculation of the relative positions of players and the distances between them (Laakso et al., 2017; Vilar et al., 2014; Winter, 2009; Duarte et al., 2010; Duarte et al., 2012). This step ensured accurate spatial measurements across all video data.

To analyze the data, a custom MATLAB (MathWorks, United States) program was developed. This program automatically identified the ball-possessing player and the nearest defensive player in each frame and measured the distance between these two players. This distance was used as a proxy for pressure, based on the principle that a shorter distance indicates higher defensive pressure exerted on the ball-possessing player. In comparison, a greater distance suggests reduced pressure and more freedom for decision-making and action. This metric allowed for a quantifiable assessment of spatiotemporal pressure during different phases of the game. Data extraction and annotation were conducted to classify actions into three categories: releasing the ball, receiving the ball, and starting a dribble. Key metrics such as success, player ID, and location were annotated. Additional subdivisions for middle and side lanes were used to provide a detailed examination of distances and interactions across these zones, helping to identify patterns of offensive and defensive pressure.

Following data extraction and annotation, the overall distance metric was represented as total D (*D*_Total_). Specific distances were recorded for key game scenarios, including the distance at the moment of ball release (*D*_Release_), the distance when receiving the ball (*D*_Receive_), the distance during successful passes (*D*_Success_), and the distance during failed passes (*D*_Fail_). Additionally, distances were measured based on the ball-handler’s position on the field: in the 30-meter goal zone (DGoal_zone_30m) and in the middle field zone (*D*_Midfield_zone_). To account for match context, distances were also recorded according to the real-time scoreline: when the team was leading (*D*_Lead_), tied (*D*_Tie_), or trailing (*D*_Behind_). This classification enabled a detailed analysis of spatiotemporal dynamics across various phases and conditions of play. To ensure data reliability, all extracted distances were manually cross-verified, and any discrepancies were resolved through a frame-by-frame review process. This comprehensive approach ensured the accuracy and robustness of the data used for subsequent analyses.

### 2.4 Statistical analysis

The data were compiled and subjected to statistical analysis using Excel 2021 and SPSS 27.0 (IBM, United States). The normality of the data was assessed using the Kolmogorov–Smirnov test. The homogeneity of variance was evaluated using Levene’s test. All data are presented as median (interquartile range, IQR). Non-parametric tests were employed to compare differences in D between Germany and China under different conditions, as well as D within the same team across different conditions. For comparisons between two groups, the Mann–Whitney *U* test was used, and for comparisons involving more than two groups, the Kruskal–Wallis *H* test was applied. *Post hoc* pairwise comparisons were conducted using Dunn’s test for multiple comparisons when significant differences were detected. Effect sizes for pairwise comparisons were reported using Cohen’s *d*. Cohen’s *d* greater than 0.8 was considered large, between 0.8 and 0.5 as medium, between 0.5 and 0.2 as small, and less than 0.2 was considered insignificant. For comparisons involving more than two groups, Cohen’s *f* was used as the effect size measure, with Cohen’s *f* greater than 0.40 considered large, between 0.40 and 0.25 as medium, between 0.25 and 0.10 as small, and less than 0.10 as insignificant. The significance level was set at *p* < 0.05.

## 3 Results

### 3.1 Differences in ball-handler distance to nearest defender between Chinese and German teams

The comparison of the overall match data reveals that the *D*_Total_ of the German team was significantly higher than that of the Chinese team (*p* < 0.001, small). Under conditions where the actual score was in favor of the team, the *D*_Lead_ of the German team was significantly higher than that of the Chinese team (*p* < 0.001, small). During instances when the score was level, the *D*_Tie_ of the German team was significantly higher than that of the Chinese team (*p* < 0.001, medium). Under conditions where the score was unfavorable, the *D*_Behind_ of the German team was significantly higher than that of the Chinese team (*p* < 0.001, medium). At the moment of ball release, the *D*_Release_ of the German team was significantly higher than that of the Chinese team (*p* < 0.001, small). At the moment of ball reception, the *D*_Receive_ of the German team was significantly higher than that of the Chinese team (*p* < 0.001, medium). Under conditions of successful passes, the *D*_Success_ of the German team was significantly higher than that of the Chinese team (*p* < 0.001, small). Under conditions of unsuccessful passes, the *D*_Fail_ of the German team was significantly higher than that of the Chinese team (*p* = 0.001, small). With the ball-handler positioned in the 30-meter goal zone, the *D*_Goal_zone_30m_ of the German team was significantly higher than that of the Chinese team (*p* < 0.001, medium). With the ball handler positioned in the middle field zone, the *D*_Midfield_zone_ of the German team was significantly higher than that of the Chinese team (*p* < 0.001, small). As shown in Table 1, the results described above are presented. While the findings are statistically significant, the small to medium effect sizes suggest varying degrees of practical impact. Medium effect sizes observed in *D*_Tie_, *D*_Behind_, and *D*_Receive_ highlight the German team’s advantage in maintaining greater spacing, which may allow for better defensive organization in dynamic or high-pressure scenarios. Conversely, the small effect sizes in metrics such as *D*_Lead_ and *D*_Success_ indicate that greater spacing is less influential when the team is already leading or executing successful passes. These results reflect contrasting defensive strategies and highlight the German team’s adaptability to different game conditions.

**Table 1:** Comparison of ball-handler distance to nearest defender between German and Chinese teams in various match situations.

Variable	GER	CHN	*z*	*p*	*d*
*D*_Total_	5.176(2.947,9.283)	3.578(2.078,5.982)	−11.944	<0.001^**^	0.463
*D*_Lead_	4.741(2.596,8.614)	3.858(2.131,6.533)	−4.279	<0.001^**^	0.250
*D*_Tie_	5.318(2.892,10.258)	2.954(1.582,4.899)	−8.101	<0.001^**^	0.696
*D*_Behind_	5.646(3.174,9.386)	3.719(2.289,5.933)	−9.162	<0.001^**^	0.595
*D*_Release_	4.245(2.499,8.042)	3.114(1.785,5.094)	−7.789	<0.001^**^	0.440
*D*_Receive_	6.334(3.600,10.711)	4.029(2.428,6.903)	−9.942	<0.001^**^	0.525
*D*_Success_	5.504(3.057,9.663)	3.731(2.164,6.235)	−11.351	<0.001^**^	0.462
*D*_Fail_	3.694(2.263,6.228)	2.828(1.685,4.506)	−3.308	0.001^**^	0.471
*D*_Goal_zone_30m_	4.105(2.420,7.807)	2.980(1.736,4.693)	−5.313	<0.001^**^	0.509
*D*_Midfield_zone_	5.615(3.073,9.828)	3.746(2.189,6.260)	−11.192	<0.001^**^	0.475

The data are presented as median (interquartile range); *z*: Mann–Whitney test statistic *z*-value; *d*: Cohen’s *d*; Cohen’s *d* greater than 0.8 was considered large, between 0.8 and 0.5 was categorized as medium, between 0.5 and 0.2 was considered small, and less than 0.2 was deemed insignificant; *p < 0.05, **p < 0.01 for differences between teams.

### 3.2 Variations in ball-handler distance to nearest defender under different conditions between Chinese and German teams

**Figure 2:**
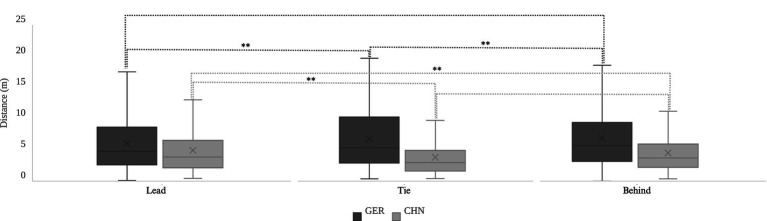
Multiple comparisons of ball-handler distance to nearest defender under three score conditions for German and Chinese teams. *p < 0.05, **p < 0.01 for differences between conditions.

The ball-handler distance to the nearest defender for the German team exhibited significant differences across conditions where the team was leading, level and trailing in the score (*p* = 0.001). The ball-handler distance to the nearest defender for the Chinese team also demonstrated significant differences across the conditions of leading, level and trailing in the score (*p* < 0.001). The *D*_Tie_ of the German team was significantly greater than the *D*_Lead_ (*p* = 0.009). However, no significant difference was observed between the *D*_Tie_ and the *D*_Behind_ (*p* = 0.460). The *D*_Behind_ of the German team was significantly greater than the *D*_Lead_ (*p* < 0.001). For the Chinese team, the *D*_Lead_ was significantly greater than the *D*_Tie_ (*p* < 0.001), and the *D*_Behind_ was also significantly greater than the *D*_Tie_ (*p* = 0.001). However, there was no significant difference between *D*_Lead_ and *D*_Behind_. As shown in Table 2, the results described above are presented. The results of the multiple comparisons are presented in Figure 2. These findings reveal tactical contrasts in defensive spacing under different score conditions. The German team maintained wider spacing during level and trailing scenarios, suggesting a focus on flexibility and counterattacking. In contrast, the Chinese team tightened spacing during level scores to limit opposition opportunities, with less variation between leading and trailing situations. These patterns highlight the teams’ differing strategies and adaptability to match dynamics.

**Table 2:** Variations in ball-handler distance to nearest defender under different score conditions for German and Chinese teams.

Teams	*D*_Lead_	*D*_Tie_	*D*_Behind_	*H*	*p*	*η*_p_^2^	*f*
GER	4.741(2.597,8.614)	5.318(2.892,10.258)	5.646(3.174,9.386)	14.254	0.001**	0.008	0.087
CHN	3.858(2.131,6.533)	2.954(1.583,4.899)	3.719(2.189,5.933)	16.368	<0.001**	0.012	0.109

The data are presented as median (interquartile range); H: Kruskal–Wallis test statistic *H*-value; *f*: Cohen’s *f*; Cohen’s *f* greater than 0.40 was considered large, between 0.40 and 0.25 was categorized as medium, between 0.25 and 0.10 was considered small, and less than 0.10 was deemed insignificant; *p < 0.05, **p < 0.01 for differences between conditions.

**Figure 3:**
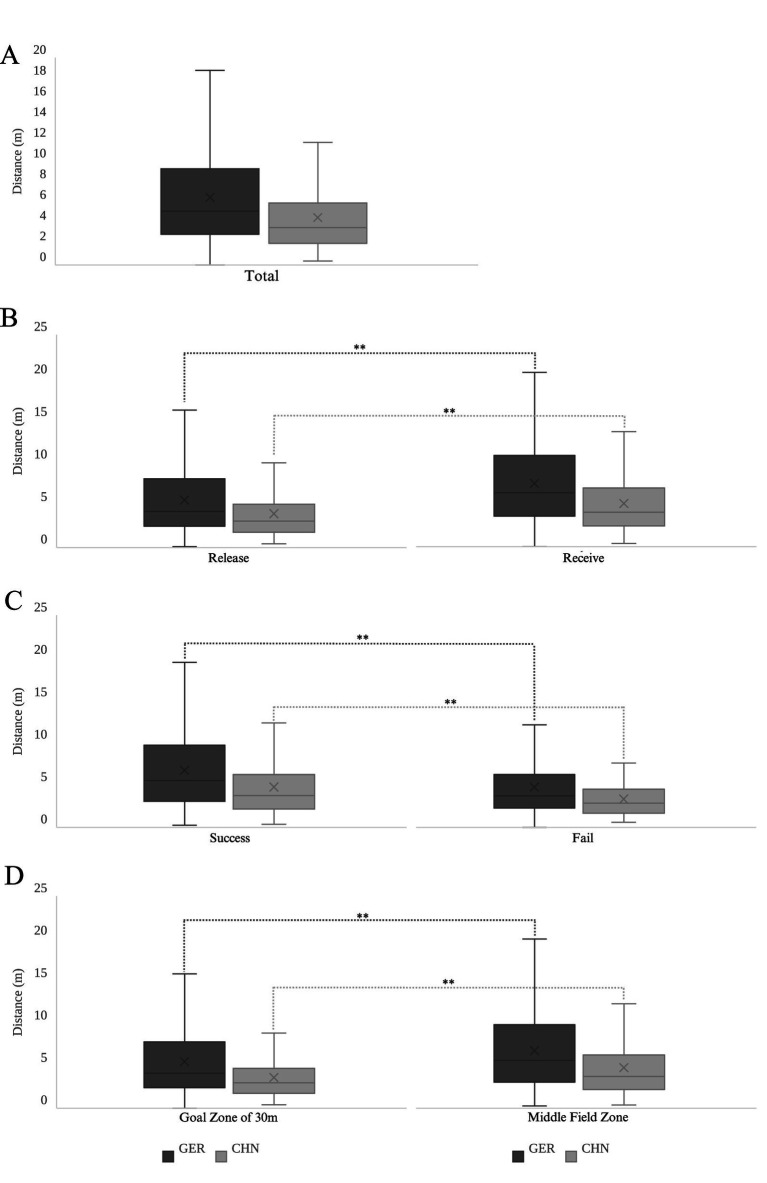
Variations in ball-handler distance to nearest defender under different conditions for German and Chinese teams. **(A)** Overall variations in ball-handler distance to nearest defender (m). **(B)** Distance variations during ball release and reception (m). **(C)** Distance variations in successful and fail passes (m). **(D)** Distance variations across different field zones (m). *p < 0.05, **p < 0.01 for differences between conditions.

For both the German and Chinese teams, the *D*_Release_ was significantly greater than the *D*_Receive_ (*p* < 0.001, Cohen’s *d* = 0.412, small; *p* < 0.001, Cohen’s *d* = 0.319, small). For the German team, the *D*_Success_ was significantly greater than the *D*_Fail_ (*p* < 0.001, Cohen’s *d* = 0.431, small); similarly, for the Chinese team, the *D*_Success_ was also significantly greater than the *D*_Fail_ (*p* < 0.001, Cohen’s *d* = 0.403, small). For the German team, the *D*_Midfield_zone_ was significantly greater than the *D*_Goal_zone_30m_ (*p* < 0.001, Cohen’s *d* = 0.281, small); likewise, for the Chinese team, a similar pattern was observed, with the *D*_Midfield_zone_ being significantly greater than the *D*_Goal_zone_30m_ (*p* < 0.001, Cohen’s *d* = 0.332, small). The variations and differences mentioned above are illustrated in Figure 3. The small effect sizes observed across these scenarios suggest that while the differences in defensive spacing are statistically significant, their practical impact may be subtle. These findings imply that the observed variations likely reflect incremental adjustments rather than fundamental shifts in tactical strategies. Such adjustments, though modest, can influence the efficiency of defensive responses, the ability to control space, and the success of transitions, particularly in high-stakes moments.

## 4 Discussion

The findings of this study demonstrate clear differences in D between Chinese and German youth football teams across various conditions. German players consistently maintained greater D compared to their Chinese counterparts, irrespective of match context or phase of play. This suggests that German players may prioritize the creation and utilization of space as a tactical advantage, reflecting superior spatial awareness and positioning strategies. Notably, under conditions where the team was leading, level, or trailing, the German team’s D was significantly greater than that of the Chinese team. Moreover, in critical phases such as ball release and reception, as well as during successful versus unsuccessful passes, the German team’s D surpassed that of the Chinese team, underscoring their capacity to generate space during both offensive actions and transitions. This spatial advantage was evident across different field zones, with midfield zones displaying greater distances compared to goal zones for both teams, albeit more prominently for the German team. These results highlight potential disparities in tactical training and match execution between the two teams.

Such findings align closely with established theories in football science, which emphasize the pivotal role of space creation and positional discipline in tactical execution. Previous studies provide valuable insights into how distance metrics contribute to both offensive and defensive strategies. Emphasizes for instance, studies have shown that players’ positional coordinates are fundamental for understanding tactical behavior, particularly in relation to the distance between attackers and defenders, as well as the space players occupy during matches (Laakso et al., 2017; Travassos et al., 2014; Silva et al., 2016). German players’ ability to maintain greater D compared to their Chinese counterparts across various conditions reflects a similar tactical approach observed in other elite teams, where spatial awareness and positional discipline are crucial in maintaining effective offensive and defensive structures (Headrick et al., 2012). In line with Laakso et al. (2017), who observed larger distances between attackers and defenders in central areas of the pitch, the German team in this study showed a consistent ability to create more space, particularly in midfield zones. This finding is consistent with the broader understanding that greater spatial dispersion allows teams to maintain offensive pressure and manage defensive transitions more effectively (Laakso et al., 2017; Headrick et al., 2012). The fact that German players were more effective in maintaining greater D during both offensive and transition phases corroborates previous research that highlights the role of distance in enhancing team dynamics during ball possession and recovery phases (Menuchi et al., 2018; Shafizadeh et al., 2016). Moreover, the consistent greater D in different match contexts—whether leading, level or trailing—further supports findings from other studies that demonstrate defensive caution and spatial reorganization as key tactics in maintaining a tactical advantage during various match phases (Duarte et al., 2012). The comparison of the two teams in this study provides further evidence that distance management is not merely a function of individual skill but also a team-wide tactical strategy that reflects deeper structural and strategic principles of the game, as also suggested by Duarte et al. (2012). Thus, the observed disparities in D between Chinese and German teams suggest differences in tactical training, particularly in how teams manage space during match play. These results further support the notion that effective space management and team cohesion can influence both individual performance and team success in football (Olthof et al., 2018; Santos et al., 2017).

These tactical differences are reflected in the distinct ways both teams manage spatial dynamics during match play. Greater D provides offensive players with more time and space to make decisions, enabling cleaner passing and more effective spatial utilization (Jankowski, 2015). This approach aligns with the German team’s consistent use of space in both midfield progression and goal zone positioning, where larger spacing facilitated controlled transitions and reduced the risk of turnovers. Defensively, a larger D reflects disciplined positioning that prioritizes structural integrity. By avoiding over-commitment, the German team effectively minimized the risks associated with close defensive engagements, such as being bypassed by quick passing or positional play. This strategic approach likely contributed to their consistent performance under varying match conditions, allowing them to adapt to different pressures while maintaining the balance between defense and attack (Rico-González et al., 2021). In contrast, the Chinese team’s smaller D suggests a focus on close defensive engagement, such as high pressing. While this approach can disrupt an opponent’s rhythm, it often leaves gaps in the defensive structure, particularly when the initial pressure is bypassed (Low et al., 2021). Offensively, the smaller D observed for the Chinese team limited their ability to create space, leading to increased defensive interference and reduced passing options (Travassos et al., 2023). These challenges underline the limitations of a smaller D in managing both defensive risks and offensive fluidity.

The observed tactical differences between the two teams are not only a reflection of match-specific strategies but also indicative of broader systemic approaches to youth development. The German team’s ability to maintain greater D and balance between offensive and defensive phases is rooted in the structural and developmental philosophies underpinning their youth football system. The German youth football system is centered on decentralized training and professional collaboration, emphasizing close coordination between youth academies, elite football schools, and the educational system (Naglo, 2020). This model has successfully produced a significant number of professional players; for instance, 80.6% of Bundesliga players born in 1993 graduated from professional youth academies (Grossmann and Lames, 2015). This structure not only ensures the professionalism of football training but also provides educational support, enabling players to balance their academic and athletic commitments. A key feature of the German training methodology is its focus on physical fitness enhancement, combined with match-scenario testing to evaluate players’ tactical performance, creativity, and game intelligence (Memmert, 2010). Compared to other European nations, Germany places greater emphasis on physical conditioning within its training programs, prioritizing the holistic development of athletic performance (Roca and Ford, 2020). This balanced training approach lays a solid foundation for players’ long-term success while fostering high levels of discipline and organization in tactical execution. The overall advantages of this model are evident in its combination of decentralized strategies, comprehensive fitness and tactical training, and the integration of informal activities, providing a sustainable framework for developing professional football talent. This system offers valuable lessons for other nations, particularly in balancing short-term performance goals with long-term development objectives.

In comparison to the German youth football system, China’s youth football development system, while making some progress in recent years, remains underdeveloped and faces numerous pressing challenges. The system comprises professional clubs, provincial and municipal sports bureaus, urban youth training centers, school football programs, and social training organizations. However, these components lack effective coordination, with each focusing predominantly on maximizing its interests. This has led to fragmented resources and insufficient integration, ultimately hindering the efficiency and quality of youth football development (Butte and Liu, 2020). Such a loosely structured ecosystem has significantly limited the potential for fostering young football talent in China.

The centralized training system in China has exacerbated these challenges, further hindering the development of a cohesive and sustainable youth football framework. Centralized training policies, such as the U23 policy, have further highlighted the systemic issues within the youth training system. Although intended to cultivate young players for the national team, these policies have had limited success and, in some cases, have even hindered player development. For instance, age-eligible players are often restricted from participating in more high-level competitions, while overage players are excluded from opportunities to progress in their careers (Butte and Liu, 2020). This short-term, results-driven approach reflects a broader issue in China’s youth football development, where the focus on immediate outcomes often overshadows the need for long-term planning (Wei, 2019). As Xiancheng (2021) research emphasizes, China’s youth football training system faces significant challenges, including a low penetration rate of football participation and a shortage of talented young players.

In contrast, the German youth football system achieves systemic coordination through a decentralized management model and close integration with the education system. This ensures a structured approach to professional training while providing players with an environment where they can balance academic and football development (Wei, 2019). Compared to the youth training systems in Japan, Spain, and France, China’s centralized training lacks a stable talent pipeline (Butte and Liu, 2020). Furthermore, the characteristics of early specialization and high training intensity in China’s youth training system increase the risk of premature career termination among elite youth football players (Li et al., 2023).

The findings suggest that greater D offers a more balanced and effective strategy, combining offensive flexibility with defensive stability. This approach not only facilitates better decision-making for ball handlers but also reduces vulnerability to opponent counterplays. For teams seeking to enhance their tactical performance, prioritizing spatial management through maintaining a large D could be a key developmental focus.

Video analysis tools, as employed in this study, can serve as a critical resource for monitoring and improving player performance. Coaches could use such tools to track player positioning and distances in real-match scenarios, offering objective feedback on tactical execution. For example, analyzing the distance between the ball-handler and the nearest defender (D) during key moments like ball release and reception can help identify patterns in spatial management. These insights can inform the design of targeted drills and training strategies aimed at improving spatial awareness, decision-making, and defensive coordination. Incorporating such data-driven methods into regular training sessions can bridge the gap between research findings and practical application, fostering long-term player development.

## 5 Limitations

The sample used in this study may not fully represent the broader characteristics of elite youth football teams in Germany and China. Factors such as team selection, competition levels, and match contexts could influence the generalisability of the findings. Expanding the sample to include diverse teams and conditions would strengthen future analyses. While D serves as a valuable proxy for assessing spatial pressure, it may not capture the full complexity of player decision-making and technical execution. Incorporating additional dimensions, such as decision quality, movement patterns, or tactical effectiveness, could provide a more holistic understanding of pressure and performance dynamics. Future research should explore the dynamic interplay between spatial pressure and performance over time, focusing on how players and teams adapt to evolving match conditions. Additionally, cross-cultural analyses of tactical development could uncover valuable insights into the relationship between training systems and spatial management strategies.

## 6 Conclusion

This study demonstrated significant differences between German and Chinese youth football teams across temporal, contextual, outcome-based, and spatial dimensions of play. The German team consistently exhibited greater D, reflecting superior spatial management and adaptability under varying match conditions. In contrast, the Chinese team’s smaller D suggests potential limitations in tactical flexibility and space utilization. These findings offer valuable insights for tactical training and youth development. For the Chinese team, enhancing spatial awareness and adaptability in training could improve performance under diverse pressures. Additionally, reducing tendencies for overly aggressive pressing, which risks defensive disorganization, may lead to more effective pressure management. Encouraging ball handlers to increase spacing in offensive phases could also support better team coordination and reduce defensive interference. However, it is important to acknowledge that the conclusions drawn from this study are based on a relatively small sample size, which may limit the generalizability of the findings. The selected matches, while representative of typical performance, may not fully capture the variability in tactical behaviors across broader competitive contexts. Future research with larger datasets and diverse match scenarios is recommended to validate these results and further explore the implications of spatiotemporal metrics in youth football training and performance.

## Data Availability

The original contributions presented in the study are included in the article/supplementary material, further inquiries can be directed to the corresponding author/s.
